# Using virtual reality simulation to address racism in a healthcare setting

**DOI:** 10.1186/s41077-024-00322-2

**Published:** 2024-12-05

**Authors:** Lindsay Beavers, Tin Vo, Julia Lee, Thanya Duvage, Hollie Mullins, Aradhana Tewari, Allison Needham, Ryan Brydges

**Affiliations:** 1 Unity Health Toronto, Toronto, Canada; 2 Department of Physical Therapy, University of Toronto, Toronto, Canada; 3 Factor-Inwentash Faculty of Social Work, University of Toronto, Toronto, Canada; 4 Wilson Centre, University of Toronto, Toronto, Canada

**Keywords:** Racism, Simulation, Virtual reality (VR)

## Abstract

Racism continues to plague Western societies’ institutions, including the healthcare system. Despite the evidence of racism’s negative impacts on healthcare providers, administrators, patients, and families, healthcare workers report hesitancy in taking action to address racism in the workplace. Simulation, with its experiential pedagogy and foundation in psychological safety, may be an educational tool to support practical training. Guided by a social cognitive view of regulation of learning, we piloted virtual reality (VR) modules focused on addressing bias, privilege, and microaggressions. We used pre-/post-surveys, reflective journals, built-in VR platform data, and simulation debriefing session notes to better understand the effectiveness and usability of these VR modules in our organization. Overall, participants found the VR modules highly valuable, and we noted a shift in participants’ reported intentions to take action to address racism in the workplace. Participants also noted the importance of a multifaceted plan that goes beyond education to ensure a meaningful culture shift toward addressing racism at work. Practical lessons from this pilot study included the necessity of an informed debriefing plan focused on participants’ positionality and power and the need to deeply understand our institution’s information technology (IT) environment to ensure successful deployment of VR technology.

**Supplementary Information:**

The online version contains supplementary material available at 10.1186/s41077-024-00322-2.

## Introduction

Racism, the social construct that certain racial groups are inferior to others [1], is firmly entrenched in the North American healthcare system and functions as a determinant of health [2–4]. Research has implicated racism in enabling healthcare practices like race correction, whereby Black patients have clinical tests “corrected” based on race, leading to late and/or misdiagnosis [5]. Multiple reviews demonstrate the devastating impacts on patients’ physical and mental health [2, 6], with racialized patients experiencing unmet healthcare needs, reduced access to care, denial of service, lack of patient safety, inappropriate hospital discharges, and negatively altered treatment decisions [7–9]. Such concerning data have led to calls for anti-racism action in healthcare [10–13]. Racism also impacts many racialized peoples’ experiences as healthcare workers through subtle and overt acts of everyday discrimination [4, 14, 15] that negatively impact their job-related and non-job-related well-being [16]. Ultimately, we believe that responsible healthcare organizations must respond to these well-known impacts of racism and the associated calls to action. We chose to use anti-racism virtual reality (VR) modules as an educational intervention with potential for addressing some concerns relating to racism in healthcare. In recognizing that anti-racism work requires attention to both desired processes and outcomes [17], this innovation paper describes our experiences planning and implementing VR modules to support anti-racism work in our organization.

### Education to combat racism

Education has long been touted as a way to address racism and its avoidable health inequities. As an example, medical school curricula have long mandated the teaching of cultural competency [18]. However, critiques of this approach include the lack of specific anti-racism training within cultural competency training [19] and the risk of reinforcing prejudices and stereotypes if the design of the curriculum does not meaningfully include those whom the education is about [20].

Approaches to teaching anti-racism in healthcare settings are notoriously heterogeneous. A recent scoping review identified commonly used theoretical frameworks and intended and unintended outcomes relating to health professional learners’ beliefs, attitudes, and behaviors [21]. Specifically, Melro and colleagues (2023) note that transformative learning and cultural safety are commonly used frameworks, which educators aim to translate into the creation of “braver spaces” [22] for dialogue and critical reflection toward enabling constructive discomfort to facilitate learning [21]. The “brave space” is a subtle shift away from “psychologically safe” [23] space and directly acknowledges the essentials of anti-racism training: power, privilege, and oppression [22]. Melro et al. (2023) also suggest that critical reflection through reflective journaling, and dialogue with peers and educators, represents key methods for anti-racism education [21]. These findings align with previous studies suggesting that two key pedagogies for teaching anti-racism include experiential learning opportunities [24] and critical reflection [25], both foundational to simulation pedagogy.

#### Role of simulation in anti-racism work

Simulation offers unique opportunities for experiential learning and, when carefully designed, for critical reflection [26]. Emerging evidence suggests that simulation training enhanced by focused debriefing could be used as a tool to address structural racism, implicit bias, and equity, diversity, and inclusion. For example, a recent scoping review of health professions simulation and equity-related content suggested that healthcare learners need training in specific behavioral skills that reduce explicit and implicit biases [27]. Notably, 87% of the reviewed simulations used standardized patients (SP) or embedded participants for role-playing [27]. While this modality choice supports realism and feedback opportunities, it also requires attending to the potential harms, such as re-traumatizing SPs who have been historically harmed by systems of oppression, reinforcing stereotypes, as well as the bias of the SPs themselves [27, 28]. Mitigation strategies to address the issues include extensive training from content experts [27, 28], which can be barriers to implementation.

As another potential solution, a recent framework adapted from transformational learning theory offers a structured approach to guide the use of simulation for equity-focused training [28]. The framework emphasizes making strategic design choices regarding the following: equipment (e.g., using racially diverse manikins), scenario content (e.g., choosing the race of the patient with the purpose of avoiding stereotyping), and people (e.g., safe involvement and training of SPs, learners, and facilitators) [28]. However, it misses some key aspects of a promising modality of simulation: VR.

VR simulations could potentially avoid some of the challenges in using simulation to address racism and promote equity through education. VR simulations allow for repeated practice and a safe(r) environment with reduced risk of SP harm [29, 30]. Avatars for VR interactions can be digitally co-created with specific patient populations and easily scaled up [31]. In-platform features can immediately provide feedback to learners and allow them to integrate it while repeating multiple scenarios [31]. Parallel to these benefits exist challenges, such as requiring additional material resources, limited efficacy for VR impacts on certain learning objectives such as psychomotor skills, and the need for additional facilitator training [29, 30]. Further, little research exists to guide how educators using VR simulation engage in the key practice of debriefing.

A core tenet of simulation debriefing involves creating a psychologically safe space [32], and, yet, we could not find literature on best debriefing practices for anti-racism training. One study using simulations to focus on racism-specific learning objectives [33] offered little explanation of the debriefing process or content. Another equity, diversity, and inclusion (EDI) and simulation-focused resource from Purdy et al. [34] is a reflective tool to prompt simulation team members to reflect on which components of their simulation included EDI and on any missed opportunities [34]. While an important guide for simulation faculty and team members, the reflective tool does not consider how to proactively address potential issues that could arise when using simulation for EDI training. Additionally, there is no guidance for the debriefing component. Understanding that VR-based simulation and debriefing are not a panacea, we looked to theory to guide our further planning to determine if the promises outweigh the concerns.

#### Informing our intervention using a social cognitive view of learning

**Fig. 1:**
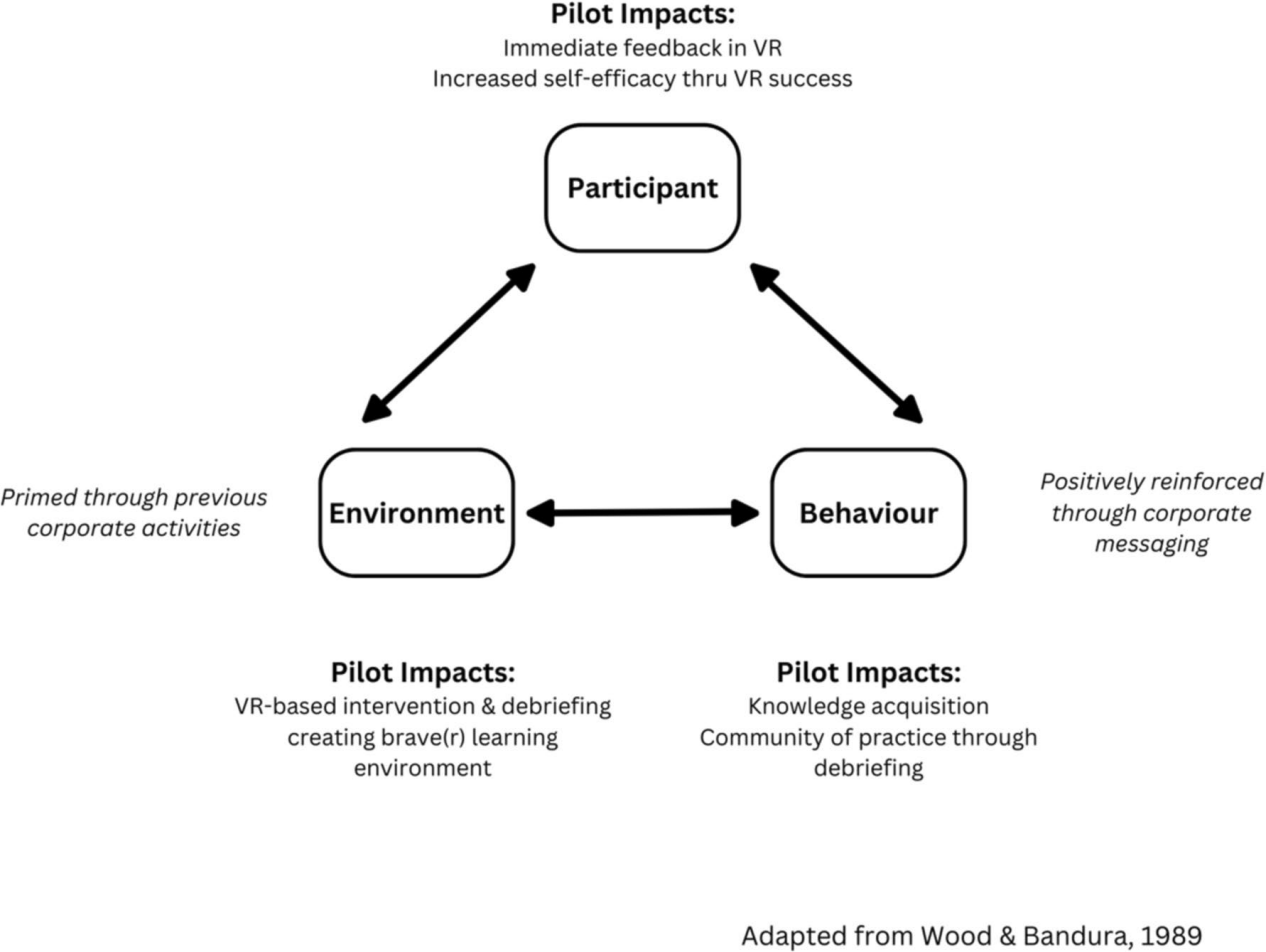
Applying the social cognitive “triadic analysis” to our proposed educational intervention processes

As we started exploring the use of VR simulations, we utilized the social cognitive view of self-regulated learning to help us consider key opportunities to enable behavior change [35–37]. According to the social cognitive view, individuals, their behaviors, and the social and physical environments in which they operate constantly interact and influence each other, such that a change in one will inevitably change the others [38]. We focused on the propositions that an individual’s self-efficacy strongly influences their behaviors, and that presenting individuals with clear expectations of the impact of change will further reinforce their desire to change behaviors [38]. Figure 1 presents how we used these principles to plan our educational intervention’s proposed processes toward our aim of enabling experiential learning and critical dialogue through debriefing and a “braver space.”

Despite the work many organizations have done to date, shifting people’s behaviors to directly address racism at work is challenging. Our leaders, for instance, self-reported that though they have received anti-racism education, they lacked confidence in addressing racism at work and desired the opportunity to practice anti-racism skills [39]. Our organization is not alone in this struggle, as evidence shows people are often cynical about engaging in discussions about race [40, 41] and often treat racism as an individual prejudice vs. a systemic issue [42]. Our team sought a better way to support people in their learning of anti-racism skills at work.

## Methods

Beyond designing the intervention, we also collected data to evaluate this preliminary pilot test of its implementation. A planning committee with expertise in anti-racism, simulation, program evaluation, and faculty development helped to develop our quasi-experimental mixed-methods pilot study. We obtained approval from the institutional review board responsible for quality improvement projects.

## Study setting

Our urban academic healthcare organization includes three main campuses with more than 11,000 staff and physicians [43]. We have had a dedicated focus on anti-racism training since 2020 as our organization started to name anti-Black racism, in part due to the murder of George Floyd and Joyce Echaquan and the long-understood disparities in health care experienced by racialized groups that were brought to the forefront through Covid-19 and resulting worldwide engagement with anti-racism work [44–47]. Corporate resources to support anti-racism work include an Office of Anti-Racism, Equity and Social Accountability (ARESA) and a council that each sets and advises executive leadership on annual accountabilities for the organization [48]. The education portfolio, which includes a state-of-the-art simulation program, partners with ARESA to support the vision for embedding anti-racism, equity, and social accountability into our culture through education and training.

### Participants

People leaders were originally targeted for recruitment; however, due to our time-limited pilot license for the VR platform (described below), enrollment was opened to all staff via email and web-based advertisements. We randomly assigned participants into two groups to help us investigate differences between the computer screen-based and full immersion versions of the VR platform.

### Intervention

We chose the Bodyswaps^©^ *Let’s Talk About Race *modules as our VR platform. These modules are intended to build knowledge and skills for talking about race. They provide opportunities to practice the skills learned and to develop readiness in recognizing and responding to power/privilege, bias, and microaggressions. Participants engaged in three modules: a) Recognizing Privilege, b) Bias as a Barrier, and c) Navigating Microaggressions.

During the pilot study, we randomized participants to two VR experiences: group A, fully immersive, using an Oculus 2 headset, wherein they could create personalized avatars (within limits) and had access to increased feedback abilities (i.e., body language) and group B computer screen-based version of the modules, which did not require a headset, still allowed for avatar creation; however, not all feedback options were available. Given the time required to complete the fully immersive version, and our participants’ constrained schedules, we opted to have participants interact mostly with the screen-based VR platform. Participants in group A completed two modules on the computer screen-based version of the VR case and one in an Oculus 2 VR headset and then immediately completed a debrief of the entire experience post VR module. By contrast, participants in group B independently completed all three modules on the computer screen-based version and then attended an in-person debrief session of their choice. Time between module completion and the debriefing session varied in group B.

#### Debriefing considerations

We used Vora et al.’s guidelines, which explicitly link psychological safety, power, and racism to inform the debriefing guide [28]. Additionally, we reflected the importance of psychological safety vs. creating a “braver space” for dialogue and constructive discomfort, in particular the acknowledgment of power, privilege, and oppression [22]. Thus, we focused additional attention on preexisting hierarchies, power and privilege from roles/disciplines, systemic racism, and each learner groups’ preexisting understanding of structural racism.

In considering the composition of our debriefing team, we chose a co-debriefing model, pairing a simulation expert with a content expert in anti-racism. Both simulation experts were full-time members of the simulation program who had extensive formal and informal training in all aspects of simulation, including in debriefing. Our content expert in anti-racism was a member of ARESA whose key institutional role involved providing anti-racism and anti-oppression expertise. We built a team with blended expertise to facilitate informal cross-training, whereby our anti-racism expert received introductory simulation debriefing training from one of the simulation experts, and the simulation experts received additional anti-racism training prior to the first session. The co-debriefers also met after each session to debrief their own experiences and identify any challenges and/or opportunities. Our debriefing team had intersecting identities, including a cis-heterosexual woman of East Asian descent (2nd-generation Canadian born to Korean-born parents), a cis-heterosexual woman of mixed Southeast Asian/South Asian descent, and a white queer cisgender woman of European descent. A co-debriefer model was also used to create a psychologically safer space for the debriefing team, knowing there would be additional power differentials with the participants who had formal leadership roles in the organization.

The debriefers sought to role model how to attend to power, privilege, and bias in the debrief session, with the goal of setting the stage for psychological safety and “braver spaces.” We purposefully planned for each participant to complete the modules independently to maximize psychological safety and not limit the number of times they could repeat a module. However, this meant the facilitators could not directly observe participants in their simulations as participants were completing them outside the simulation lab. Those who completed the modules in the Oculus headsets did not have their experience cast out to the larger room or directly observed by the facilitators. Thus, the debriefers had no ability to ask reflective questions based on directly observed behaviors, resulting in the need for a comprehensive debriefing guide.

We adapted our guide based on the *Promoting Excellence and Reflective Learning in Simulation* (PEARLS) debriefing framework [49]. The adaptations focused most on the reactions phase, as we aimed to normalize learning challenges, build a sense of common struggle, and to further enable a “braver space.” The guide’s questions in the description and analysis phases were selected to match the modules’ learning objectives, as we sought to have participants reflect on areas where they felt they were successful and areas for continued growth while encouraging self-reflection on social locations and resulting power and privilege. The debrief wrapped up with some directive teaching of resources available to further participants’ learning.

#### Data collection

Pre-intervention, we used a customized online survey to collect demographic data. Given the pilot nature of the work and the likely low number of recruited participants, we collected minimal demographics with a focus on who was engaging with the opportunity. We distributed a pre-intervention survey asking participants to provide a reflective journal entry discussing a situation, observation, or issues they had experienced or witnessed in the past month related to racism manifesting through power/privilege, bias, and/or microaggression.

Seven days post-intervention, we distributed a survey to collect participant’s key takeaways from the education modules; their self-assessment of readiness to recognize and discuss racism and issues of power/privilege, microaggressions, and/or bias incidents in the workplace/care environment; and their perceived supports needed to enhance their readiness to recognize and respond (i.e., readiness to change). Additionally, participants submitted a post-intervention reflection journal entry on the same scenario as before, i.e., about a situation when issues related to race/racism might have arisen or a situation, observation, or experience that they might have had in the past month that related to power/privilege, bias, or microaggression. Participants had up to 30 days to complete the post-intervention survey and reflection. Additional File 2 includes copies of the pre-post online surveys.

Other data collection included in-platform data for time participants spent in the module, simulation program team time supporting the pilot’s technical aspects, and the debrief facilitators’ notes taken during each debrief session. Midway through the pilot, we extended our VR platform subscription to 60 days to support additional recruitment and expanded our inclusion criteria to any staff vs. only formal leaders.

#### Data analysis

We used descriptive statistics to analyze participants’ responses to the pre- and post-intervention surveys. Two team members each thematically analyzed the pre-post journal entries within groups A (L. B. & A. W.) and B (J. D. & T. V.), and then each dyad met to discuss overarching themes, according to principles of reflexive thematic analysis [50]. Team members used MS Excel and Word to generate, catalogue, and summarize the themes. The debriefing sessions were not recorded; however, session notes were taken and thematically analyzed by three team members (L. B., J. D., & T. D.). The post-intervention readiness to change questions was analyzed by two team members (L. B. & H. M.) and was used to supplement the understanding of the journal entries and debriefing notes.

For all thematic analyses, we conducted deductive analyses using the module objectives to support the development of an initial coding framework. We also engaged in inductive analyses by attending to emergent codes that each team member identified during their analyses. We met regularly as a team to discuss our analytic frames, to challenge each other’s emerging codes, to select a final coding structure, and to enhance the comprehensiveness of the analyses [51].

## Findings and discussion

We ran a total of eight simulation and debriefing sessions over the course of 2 months. Each site had at least one session, and, on average, about four participants attended each session.

### Demographics

Table 1 presents participants’ demographic data across the three sites. We did not conduct any statistical analyses of these data and judged them as showing that the two groups had reasonable similarities in their professional and personal characteristics.

**Table 1:** Participant demographic data

Participant demographics	Total (*n* = 33)	Group A (*n* = 15)	Group B (*n* = 18)
Position/role			
People leader	78.8%	80.0%	77.8%
Prefer not to say	21.2%	20.0%	22.2%
Primary site of work			
Site no. 1	60.6%	46.7%	72.2%
Site no. 2	18.2%	13.3%	22.2%
Site no. 3	15.2%	33.3%	0.0%
Prefer not to say	3.0%	0.0%	5.6%
No response	3.0%	6.7%	0.0%
Participants identifying as a racialized person and/or as belonging to a racialized community?			
Yes	30.3%	33.3%	27.8%
No	69.7%	66.7%	72.2%

### Usability data

We collected the average length of time each group spent in each module (Table 2), with no meaningful difference between the groups. Both groups engaged in the bias module for the longest amount of time and the microaggressions module the least.

**Table 2:** Time spent in VR per module

Module	Group A average	Group B average	Overall average
Bias	41 min	38 min	39.5 min
Privilege	32 min	32 min	32 min
Microaggressions	27 min	31 min	29 min

We also collected data to understand the impacts of using VR simulation on the simulation program team’s resources. Our administrative support took 5 h to set up participants in the portal and to pull the “time spent in VR per module” data. It took just over 1 h to set up each VR session for a total of 5 h of setup. Notably, there were technology issues, and two team members spent a combined 8-h troubleshooting before and during the VR sessions. The main feasibility lessons we learned relate to how to integrate the VR platforms into our organization’s information technology (IT) network.

In using a third-party platform, the licensing limits meant that our pilot testing needed to be done based on an accelerated timeline of 30 days. We did extend the license for another 30 days at an additional cost. While this cost was covered, a preexisting negotiation with the VR platform developer for extensions could have reduced the financial burden and increased our final participant numbers.

We also did not predict the challenges of running a VR platform in a hospital IT network environment. Our hospital IT network is designed with low-risk tolerance to protect sensitive patient data, meaning the system does not allow for substantial data packages to be downloaded without planning and intervention by IT specialists. As a workaround, we often had to rely on creating our own hotspots via our phones to allow the VR platform to run smoothly. Conversely, we easily incorporated the computer screen-based version into our IT network. We proactively worked with our IT colleagues before launch to deem it safe and package it as a download that was available to staff with no administrative login required. This work made it seamless for participants to engage in the modules with minimal study team resources.

#### Effectiveness data

Our pre-post survey data and notes on the debriefing sessions helped us to understand how the VR modules impacted learners’ experiences and provided some data on how the fully immersive experience compared to the computer screen-based version. In group A, 12/15 (80%) responded to the post-survey, though only 7/15 (47%) fully completed it, including the post-reflection journal entry. For group B, 14/18 (78%) responded, though only 8/18 (44%) completed the post-reflection journal entry. Overall, the data suggested that participants reported minimal impacts of the modality on their experiences or time spent in the modules, offering preliminary data suggesting that participants perceived similar user experiences between the VR headset and computer screen-based versions.

In terms of participants’ experiences, survey data analysis identified three themes: (a) hesitancy for action, (b) types of responses, and (c) enablers for action. Participants expressed a *hesitancy for action*, which reinforced previously established organizational knowledge that there is hesitancy in addressing racism at work. As a participant noted, “‘…although I was uncomfortable with the incident, I did not speak against the comments that were said. I stayed silent…I was not sure about how to proceed in this situation.’ –P28.” The existence of multiple reports and guidance documents for organizations to address racism demonstrates that this hesitancy is not individual, rather appearing to be an issue across systems [52–54].

Our analysis suggested the *types of responses* theme showed a shift toward action, with participants feeling ready to act to address racism after completing the modules. As one participant noted, “‘armed with the techniques from my recent training, I was able to react [to the situation] in a way that allowed me to call out the behaviour, instead of being a passive bystander.’-P7.” These findings align with Medvec et al. [55], who used immersive VR to support EDI and anti-racism training in nurses with positive results.[55].

In highlighting *enablers for action*, participants reported highly valuing the modules and noted that they would only be effective if our organization continued to take steps to foster a culture that supported individuals to take action to address racism, “‘the workplace needs to make it extremely clear that it’s okay and safe for staff to recognize incidents surrounding microaggressions, bias, privilege, etc.’. –P25.” These reflections align with psychological safety literature whereby people need to feel psychologically safe in the workplace before they act to apply their knowledge and engage in any transformative change [23, 56].

Our two themes produced from analyzing the reflective journals also aligned with the debriefing analysis data: (a) feelings and (b) future work. Participants in both groups had strong and negative feelings going through the modules, with the most common descriptions of participants’ experiences being “awkward, discomfort, anxiety, and embarrassment.” The debriefing analysis also identified a tension between participants’ descriptions of the modules as being “easy” or “basic” and their feelings. That participants could explicitly label their negative experiences might be a sign that our efforts to create a “braver space” in the debriefing sessions were successful. Moreover, despite the negative emotions, participants all noted that anti-racism conversations are important, and that future work within our organization is needed.

#### Study limitations

While we maximized our data collection within this pilot’s scope, we were limited in long-term follow-up. We collected numerous effectiveness and usability measures; however, our follow-up with participants was up to 1-month post-intervention. Exploring the longer-term impact of this educational intervention would be more effective in understanding if there was meaningful, sustained behavior change. Further research examining such sustained behavior changes would be beneficial, as would recruitment of a larger sample size.

## Conclusions

Organizations and individuals are taking steps toward dismantling structural and interpersonal racism in healthcare, and the use of VR simulation, in fully immersive and screen-based formats, may offer a unique educational intervention to help shift behaviors. Our pilot study sought to use VR simulations to support whether and how our leaders and staff address racism in the workplace. Our findings showed promise in that both immersive and screen-based VR versions showed potential as educational interventions to support participants in learning to take action to address racism. For usability, we found that using a VR platform does take considerably more resources than other simulation approaches, which may be a barrier for some institutions. Notably, we had the privilege of access to both anti-racism and simulation content experts, which were key enablers to study completion. We believe the VR modules could be successfully delivered together with education and other tools (e.g., quick tips on lanyards), enabling “hands-on” practice to create a safety and learning culture that empowers staff to talk about racism.

## Data Availability

Aggregate pre- and post-survey results, and the analysis of the debriefing notes, are available upon request.

## Appendix

Additional file 1: Fig. 1. Applying the Social Cognitive ‘triadic analysis’ to our Proposed Educational Intervention. Additional file 2. Pre_Post Survey Questions.

## Abbreviations

VR: Virtual reality
ARESA: Office of Anti-Racism, Equity and Social Accountability
EDI: Equity, diversity, and inclusion
SP: Standardized patients
PEARLS: Promoting Excellence and Reflective Learning in Simulation
IT: Information technology
