# Supplementary figures and images for: Using virtual reality simulation to address racism in a healthcare setting
Source: Adv Simul (Lond). 2024 Dec 5;9:46. doi: 10.1186/s41077-024-00322-2 (PMC11622567; doi:10.1186/s41077-024-00322-2)

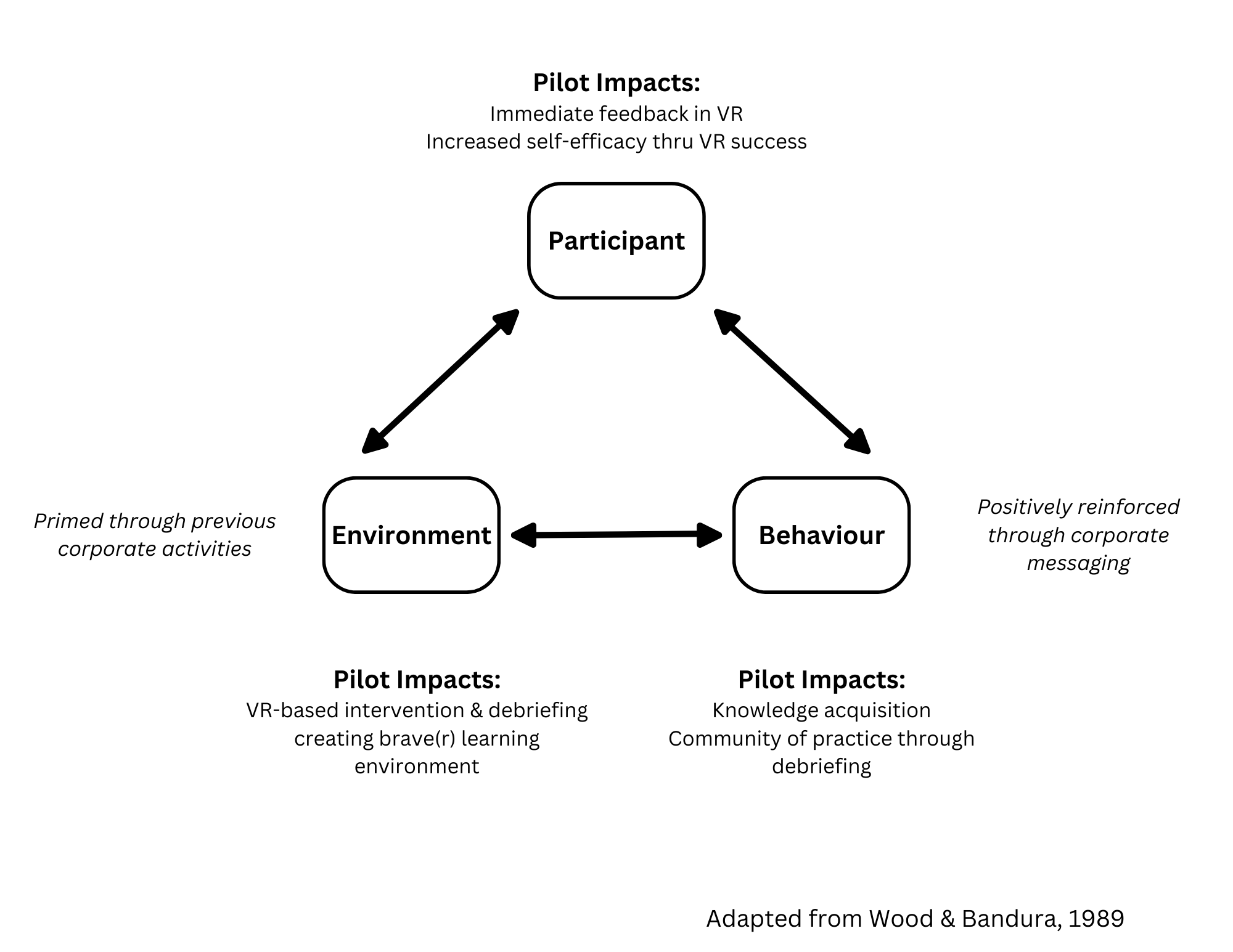

Supplement: Supplementary file 1 — Additional file 1: Fig. 1. Applying the Social Cognitive ‘triadic analysis’ to our Proposed Educational Intervention. [file 41077_2024_322_MOESM1_ESM.png]
