# Supplementary material for: Using virtual reality simulation to address racism in a healthcare setting
Source: Adv Simul (Lond). 2024 Dec 5;9:46. doi: 10.1186/s41077-024-00322-2 (PMC11622567; doi:10.1186/s41077-024-00322-2)
Supplement: Supplementary file 2 — Additional file 2. Pre_Post Survey Questions. [file 41077_2024_322_MOESM2_ESM.pdf]

Pre-Intervention Online Survey

Q1. What is your name? [open text]

Q2. What is your email address? [open text]

Q3. What is your current role [options: People Leader, Physician Leader, Prefer not to say]

Q4. What is your primary site of work [options: the three sites, plus Prefer not to say]

Q4. Do you identify as a racialized person and/or as belonging to a racialized community? [options: Yes, No, Prefer not to answer]

Q5. Reflective journal entry. Think about an incident related to bias or microaggression that you might have witnessed in the past month. Briefly describe the incident, keeping it at a high level to avoid any potentially identifying information.

Reflect on the following questions based on the scenario provided above, or the incident you described:

1. What were your positionalities/social location in relation to the perpetrator and the victim?
2. How did the incident make you feel? What kinds of feelings arose? Could you describe or explain those feelings?
3. What did you think about the incident? What thoughts went through your mind? How did you process those thoughts?
4. How did you react to the incident? How did you handle the incident? What did you do?

Please note: If you need to talk to a mental health professional regarding any of the above reflections, you are asked to follow-up with the Employee Assistance Program, or your own mental health professional.

Post-Intervention Online Survey

Q1. Based on what you learned in the three modules, what practice(s) did you implement in the workplace? What behavior(s) did you change? [Open text]

Q2. To what extent do you feel ready to recognize issues of power/privilege, microaggressions and/or bias incidents in the workplace? [options: Not at all ready, Somewhat ready, Very much ready]

Q3. To what extent do you feel ready to respond to issues of power/privilege, microaggressions and/or bias incidents in the workplace? [options: Not at all ready, Somewhat ready, Very much ready]

Q4. Please explain your response in terms of readiness to recognize and respond to issues of power/privilege, microaggressions and/or bias incidents in the workplace. [OPEN TEXT]

Q5. What else do you need to support you in recognizing power/privilege, microaggressions and/or bias incidents in the workplace, as well as responding to them? This can include training, practice, and another other supports that might be helpful. [OPEN TEXT]

Q6. Reflection Journal Entry. Think about an incident related to bias or microaggression that you might have witnessed in the past month. Briefly describe the incident, keeping it at a high level to avoid any potentially identifying information.

Reflect on the following questions based on the scenario provided above, or the incident you described:

1. What were your positionalities/social location in relation to the perpetrator and the victim?
2. How did the incident make you feel? What kinds of feelings arose? Could you describe or explain those feelings?
3. What did you think about the incident? What thoughts went through your mind? How did you process those thoughts?
4. How did you react to the incident? How did you handle the incident? What did you do?

Please note: If you need to talk to a mental health professional regarding any of the above reflections, you are asked to follow-up with the Employee Assistance Program, or your own mental health professional.
